# On the static structural design of climbing robots: part 2

**DOI:** 10.1186/s40638-015-0031-x

**Published:** 2015-11-25

**Authors:** Ausama Hadi Ahmed, Carlo Menon

**Affiliations:** MENRVA Research Group, School of Engineering Science, Simon Fraser University, Burnaby, BC V5A1S6 Canada

**Keywords:** Climbing robot, Geometry design, FEM, Biomimicry

## Abstract

This manuscript is the second of two parts of a work investigating optimal configurations of legged climbing robots while loitering on vertical surfaces. In this Part 2, a structural analysis based on the finite element method, specifically the stiffness method, is performed to address the problem. Parameters that are investigated in this Part 2 include the inclination of both the body and the legs of the robot. Outcomes of the performed study are validated by analyzing the posture of 150 ants when loitering on vertical surfaces. The obtained validation ensures the predictions of the developed structural model are correct and can be used to identify optimal configurations of legged robots when loitering on vertical surfaces.

## Background

Climbing robots have a wide range of potential applications such as inspecting airplane wings, bridges and wind turbine blades, cleaning sky scrapers, welding and painting ships and tanks, maintaining nuclear plants, and in agriculture, surveillance and security. The structural design of some climbing robots developed by the scientific community was inspired by nature’s living organisms including worms [1], spiders [2], geckos [3], cockroaches [4] or a combination of different species [5].

Climbing robots have been designed to adhere to climbing surfaces using different mechanisms such as magnets [6, 7], electrostatics [8, 9] and air vortex [10, 11]. A number of the attaching mechanisms also mimic the nature. For instance, Sky Cleaner IV [12] and Robicen III [13] use suction to adhere to the climbing surfaces mimicking the suction cups of the octopus; Stikybot [14], Mini-Whegs [15], Waalbot [16] and Abigaille [2, 17] use dry adhesives inspired by geckos; and Spinybot [18], and RiSE [19] use hooks inspired by cockroaches to adhere to non-smooth surfaces.

In this work, the effect of different geometrical parameters on the maximum attachment force required by a robot to stay attached to a vertical surface is investigated. Specifically, the optimal configuration is considered to be the one that minimizes the adhesion requirements for the robot, that is minimizes the minimum of the maximum adhesion force of any foot.

In the first part (Part 1 [20]) of this two-part work, the body of the robot is assumed to be perpendicular to the legs and parallel to the climbing surface. Dimensions are normalized, that is, they are divided by the distance between the front and hind feet of the robot. Main findings of Part 1 are: the body height to body length ratio should be as small as possible; the normalized middle leg position should to be between 0.24 and 0.41 where the middle leg is positioned at 0 when it overlaps the hind leg and at 1 when it overlaps the front leg; and the stiffness of the body should be much larger than the one of the legs (e.g., the thickness of the body should be larger than that of the legs).

In this second part (Part 2) of the work, the role of both the inclination of the body and legs is investigated using the finite element method (FEM) [21]. The investigated parameters provide guidelines to design robots that loiter on vertical surfaces with minimum normal adhesion force. In addition, an example from nature is investigated to validate the assumptions and simplifications used in the developed FEM model. Specifically, the posture of ants when they are on vertical surfaces is investigated. An experiment is carried out to measure the geometrical parameters of the ants’ postures while on vertical surfaces. Many researchers have studied the stepping patterns in ants under the influence of speed [22, 23], curvature [23], different body geometries [24], under the influence of different loads [25] and on different slopes [26]. To the best of the authors ‘knowledge, little work was, however, performed on ants’ postures while loitering on vertical surfaces.

This paper is organized as follows. “Investigated parameters” section completes the theoretical investigation of Part 1. Specifically, the inclination of both the body and the legs are investigated. “Model verification: structural analysis for ants’ stance on vertical surfaces” section presents a verification of the performed two-dimensional FEM analysis. Specifically, predicted results are compared to postures ants have on vertical surfaces. Conclusions and recommendations for the design of legged robots operating on vertical surfaces are presented in “Discussion” and “Conclusion” sections.

## Investigated parameters

**Fig. 1:**
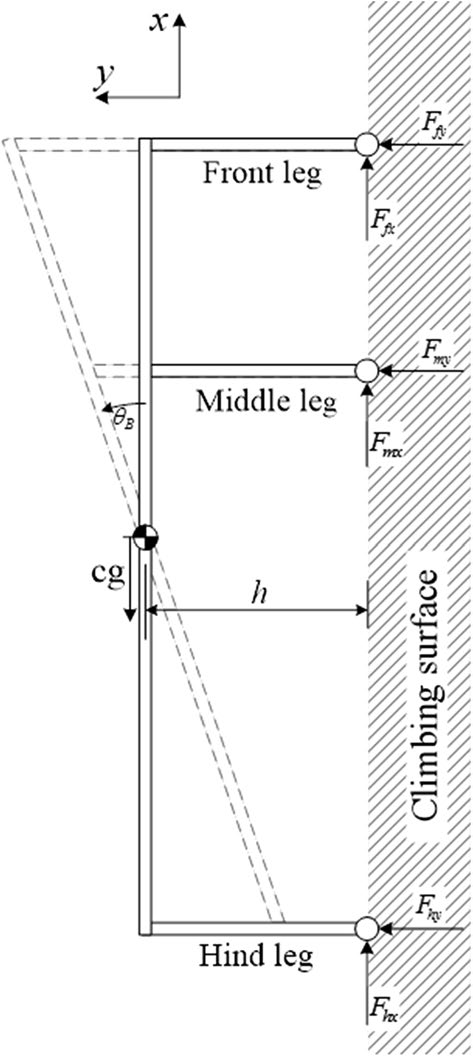
Robotic structure with an inclination angle of *θ*_B_

In this section, a six-legged robot is studied. Due to the symmetry of most legged robots, a two-dimensional analysis was considered to be suitable [20]. The maximum of the normal adhesion forces on the tips on the legs, i.e., $F_{\text{fy}} , F_{\text{my}}$ and $F_{\text{hy}}$ in Fig. 1, required by the robot to stay attached to a vertical surface is minimized by examining different parameters of the structure using the FEM model developed in Part 1. The investigated parameters are: (1) the position of both the middle leg and (2) the body’s and (3) the legs’ inclination. These parameters are defined and investigated in the following sub-sections.

### Body inclination and middle leg’s position

Similar to Part 1, the robot in this paragraph is assumed to have a height to body length ratio of 1:2. Results drawn from this specific geometry are generalized in a subsequent section.

Using a robot with height to body length ratio of 1:2 implies that collision occurs when the body inclination is either over 45° or less than −45°, where 0° inclination is defined when the robot’s body is parallel to the climbing surface (see angle $\theta_{\text{B}}$ in Fig. 1). The height and the distance between the front and the hind legs are considered to be fixed to keep the height to length ratio fixed at every inclination, see Fig. 1. As discussed in Part 1, no units are used, as the results can be scaled up/down.

**Fig. 2:**
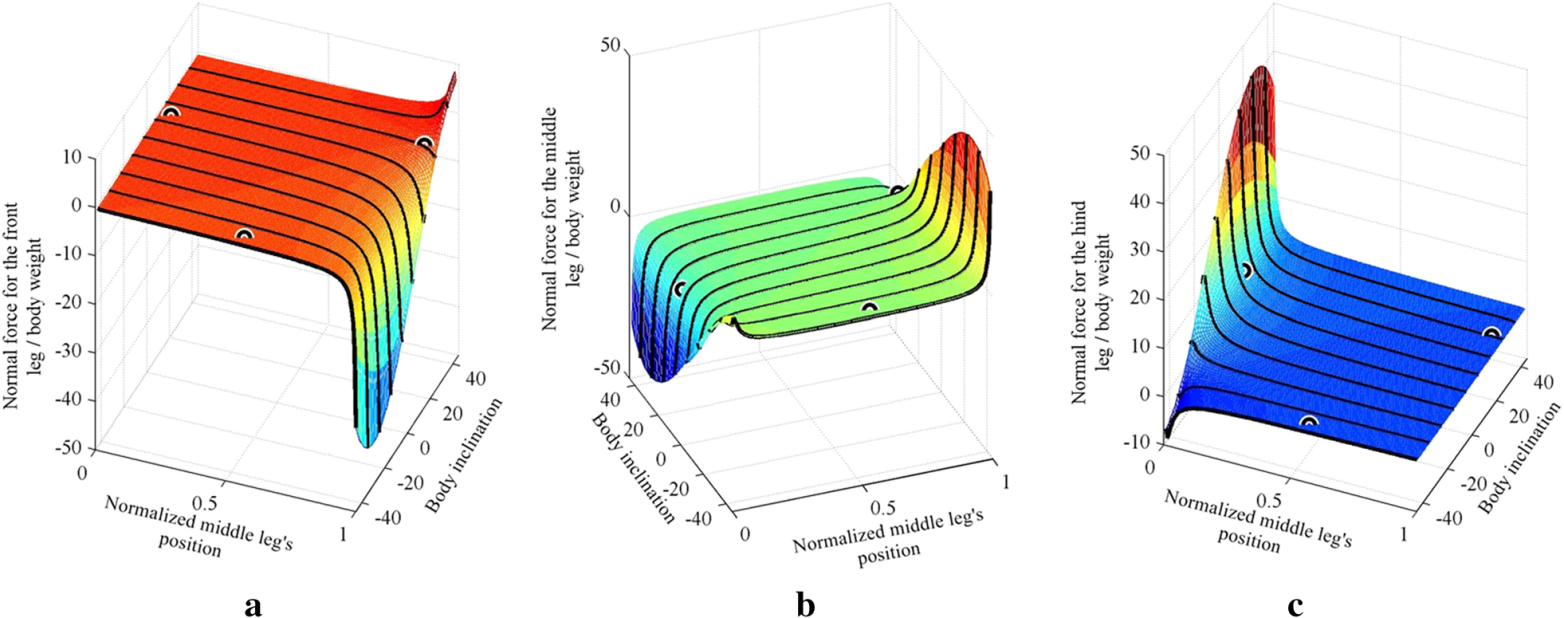
The normal forces for different body angles and different middle leg’s positions for **a** front leg, **b** middle leg, **c** hind leg. *Circles* on the plot represent simulations performed using ANSYS

The effect of changing both the position of the middle leg and the inclination of the body is shown in Fig. 2. Three different configurations are compared with ANSYS and plotted (see circles) over the curve obtained using MATLAB in Fig. 2, with an average error of approximately 1.29 %.

**Fig. 3:**
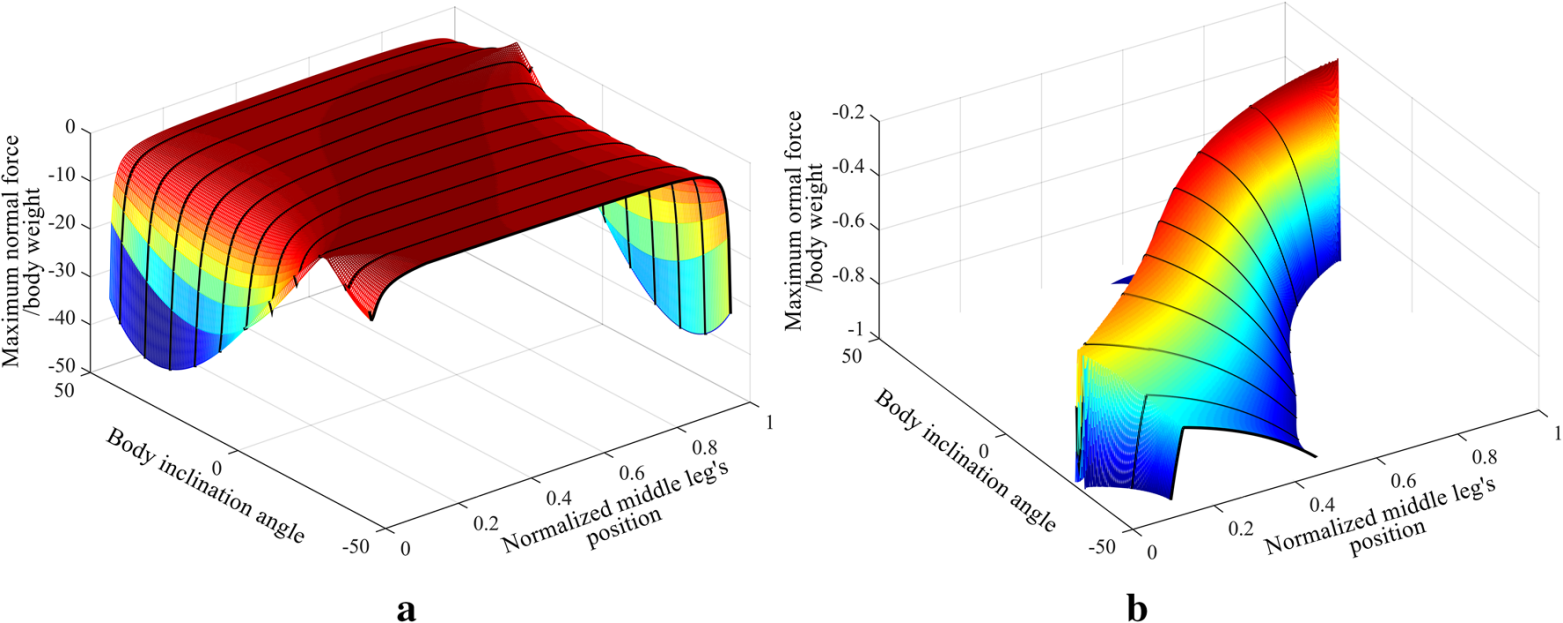
**a** Shows the maximum adhesion force required by any foot for different body angles and different middle leg positions** b ** is a zoom-in showing the behavior of the curve near its optimal point

The maximum adhesion force required by each leg for the different angles of the body is shown in Fig. 3. The optimal structure in Fig. 3 is found to have a body angle of approximately 28.25° and a middle leg’s position of 0.99, where the middle leg’s position is bounded in the 0.01–0.99 range.

The inclination of the body by a positive angle $\theta_{\text{B}}$ (counterclockwise angle in Fig. 2) causes the beams of the front half body of the robot to become longer (see Fig. 2) and therefore more flexible, whereas the beams on the hind half body (see Fig. 2) to become shorter and therefore stiffer. A negative body inclination angle causes an opposite effect. In other words, the body inclination affects the stiffness of the different parts of the robot. This general behavior can be generalized to robots having different height to length ratios. It should be noted that a robotic structure with a smaller height to length aspect ratio would have a smaller inclination range, thus limiting the choice of optimal body inclinations.

### Leg’s inclination angle and middle leg’s position

The effect of having the legs inclined instead of perpendicular (see Part 1) to the climbing surface is investigated in this section. Similar to the previous sections, the adhesion force required to keep the robot on a perpendicular surface is assumed to be minimized.

**Fig. 4:**
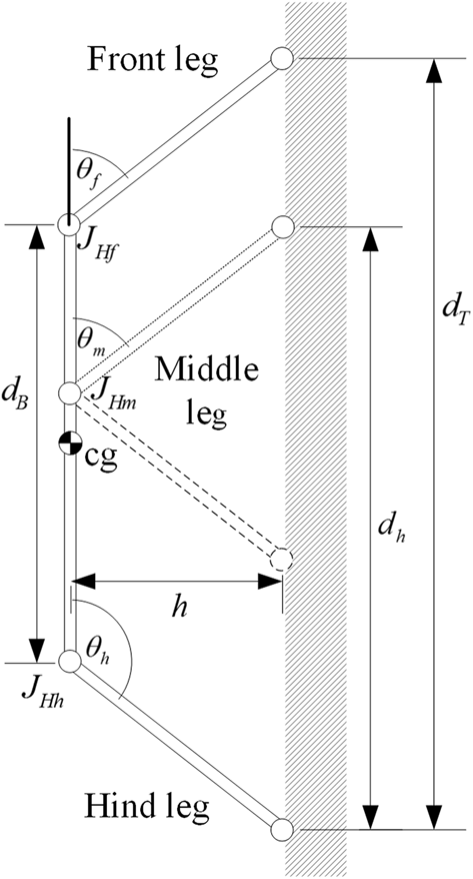
The robot with the inclined legs

The robot’s structure is the same as that used in the previous section (a body length of 200 and a height of 100). The legs are arranged so they are inclined outward, i.e., the front leg is inclined forward and the hind leg is inclined backward to mimic climbing arthropods such as ants, cockroaches, and spiders. In this section, two cases for the middle leg are considered. In the first case, the middle leg is inclined towards the front of the robot with inclination angle equals to the front leg’s inclination (see the solid lines in Fig. 4). In the second case, the middle leg is inclined towards the back of the robot with inclination angle equals to the hind leg’s inclination (see dashed lines in Fig. 4). Figure 4 shows a diagram of a robot, where $d_{\text{B}}$ is the length of the body, *d*_T_ is the distance between the tip of the front leg and the tip of the hind leg which is kept fixed at 200, and *θ*_f_, *θ*_m_ and *θ*_h_, the angles the front, the middle, and the hind legs make, respectively, with the body.

The range of the front leg’s angle is from −90° to −45°, and the hind leg’s angle to range from −135° to −90°. At the maximum inclination, i.e., *θ*_f_ = *θ*_m_ = −45° and *θ*_h_ = −135°, all of the three joints are located at the center of mass. A wider inclination range at this height is not feasible without increasing the distance between the tips of the front and the hind legs.

The case when the middle leg’s inclination equals the front leg’s is considered first. An inclination angle *θ*_inc_ is introduced to represent the inclination of the legs. All the legs are perpendicular to the climbing surface at *θ*_inc_ = 0°, and the front and the middle legs have a −45° angle with the body and the hind leg has −135° with the body at *θ*_inc_ = 45°. The angle of the front and the middle legs can be represented using the following equation:

1 $$\theta_{\text{f}} = \theta_{\text{m}} = - 90 + \theta_{\text{inc}}$$

The hind leg’s angle can be represented as a function of *θ*_inc_ as follows:

2 $$\theta_{\text{h}} = - 90 - \theta_{\text{inc}}$$

**Fig. 5:**
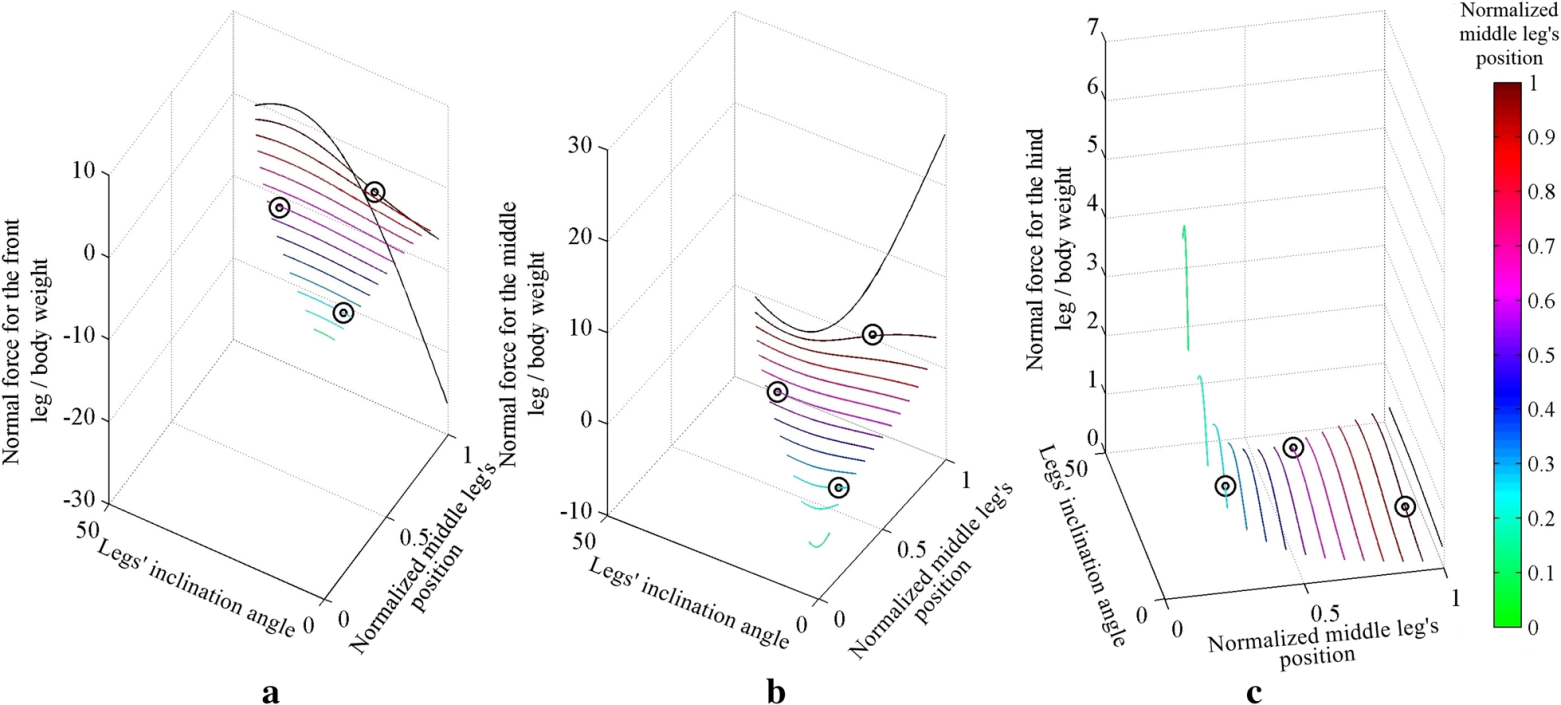
The normal force distribution with the middle leg inclines forward for different *θ*_inc_ values and different middle legs’ positions, on **a** the front leg, **b** the middle leg and **c** the hind leg. *Circles* represent simulations performed using ANSYS

The normal force distribution for the range of *θ*_inc_ with different middle leg positions is shown in Fig. 5. Three different configurations are compared with ANSYS and plotted over the curve obtained using MATLAB, with an average error of approximately 0.14 %. The position of the middle leg’s joint (*J*_Hm_) is represented as a fraction of the distance *d*_T_ where the value 0 is positioned at the hind leg’s tip and 1 being at the front leg’s tip. The curves of the different legs’ positions have different lengths, because the range available for the hip position of the middle leg decreases as the angle *θ*_inc_ increases.

**Fig. 6:**
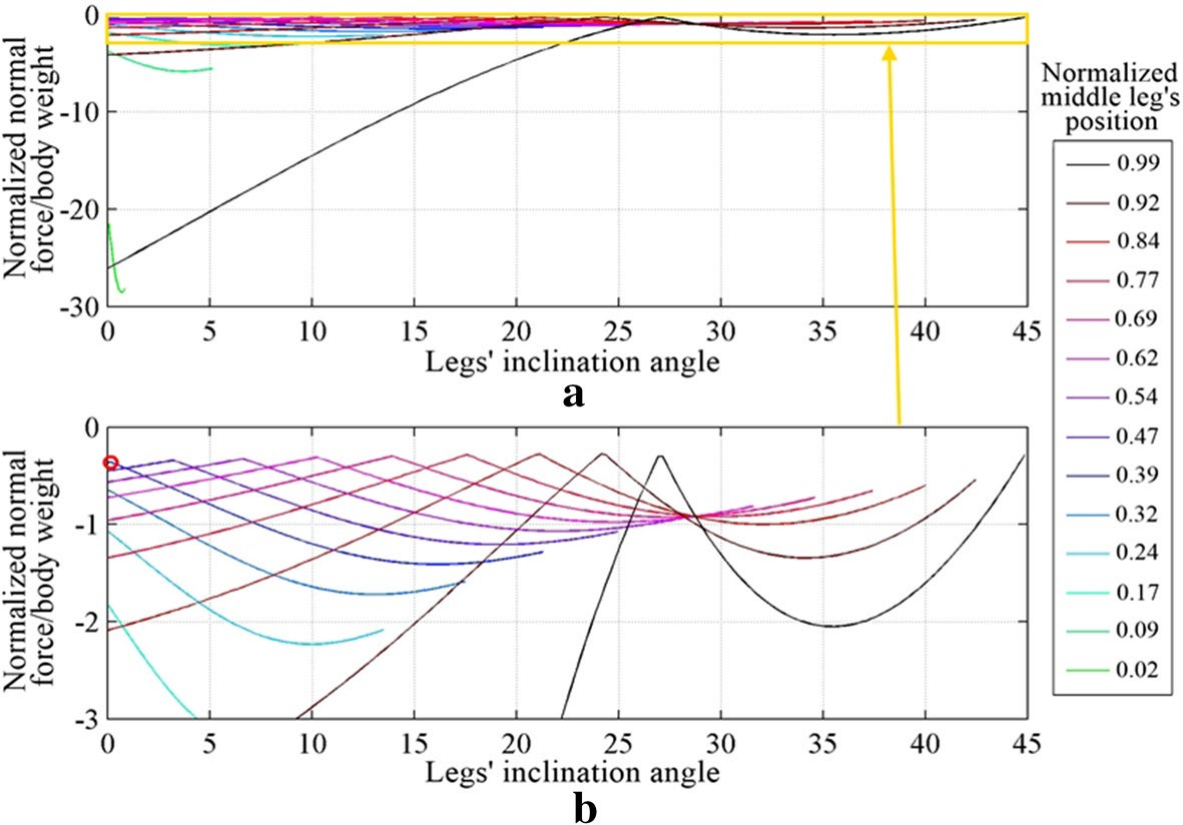
**a** The maximum adhesion required by the robot at different legs’ inclinations and different middle leg’s positions. **b** Zoomed in view of plot (**a**). The *red circle* in (**b**) shows the closest middle leg’s position to the hind leg that improves with forward inclination of the middle leg

The maximum adhesion force applied by the robot to the vertical surface can be identified by analyzing Fig. 6, which combines the three subplots of Fig. 5. It can be noted that inclining the middle leg forward improves the adhesion requirement when the position of the middle leg is greater than 0.38 (see red circle and legend in Fig. 6b). Bringing the middle leg closer to the front leg, with the optimal inclination for that position, results in requiring less adhesion force.

**Fig. 7:**
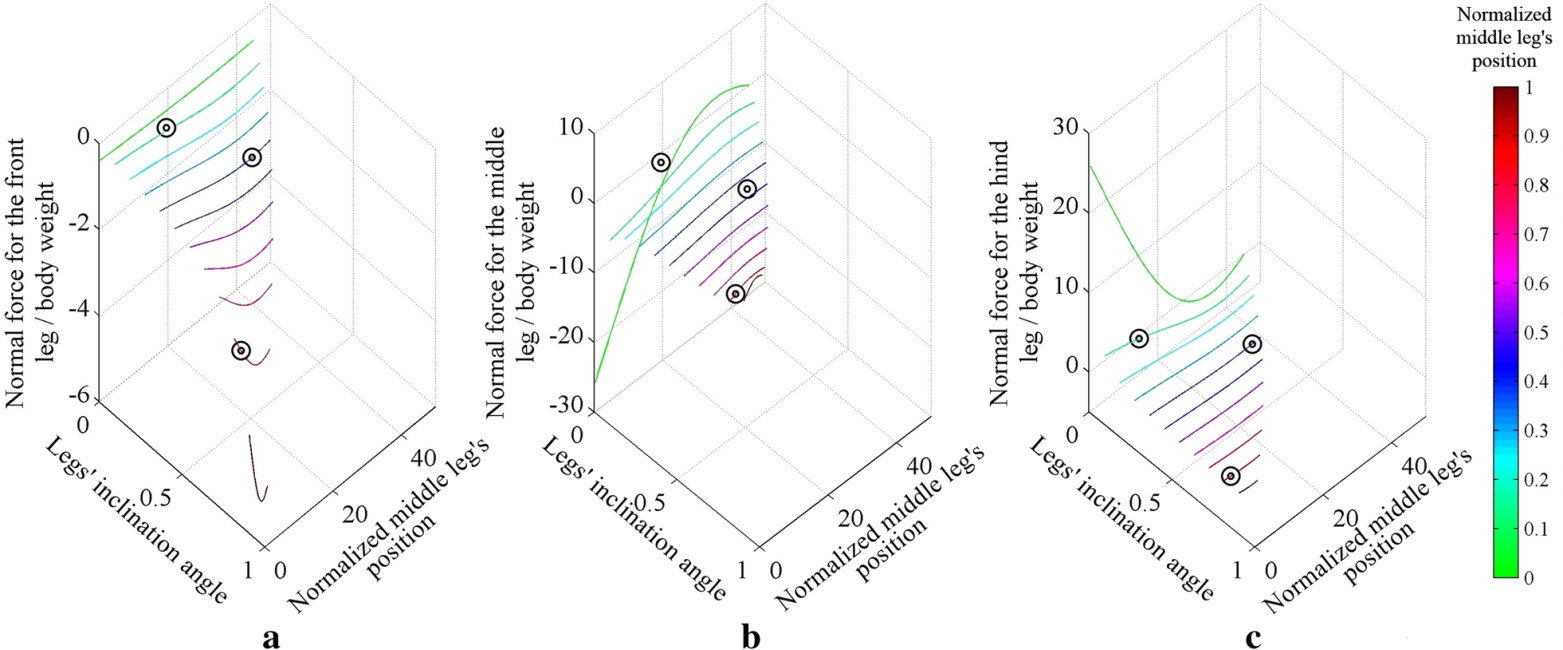
The normal force distribution on the legs of the robot for different legs’ inclinations and different middle legs’ positions on **a** the front leg, **b** the middle leg and **c** the hind leg, with the middle leg inclined backward. *Circles* represent simulations performed using ANSYS

The case when the middle leg inclines backward, i.e., has the same inclination angle as the hind leg’s angle, is investigated similar to the previous case; an inclination angle *θ*_inc_ is used to represent the inclination of the legs. The equations of the inclination angle for both the front leg, Eq. 1, and the hind leg, Eq. 2, are the same as in the previous case; while the inclination angle for the middle leg is the same as the hind leg’s equation angle, i.e., Eq. 2. The normal force distribution for the range of *θ*_inc_ with different middle leg positions is shown in Fig. 7. Three different configurations are compared with ANSYS and plotted over the curve obtained using MATLAB, with an average error of approximately 0.13 %.

**Fig. 8:**
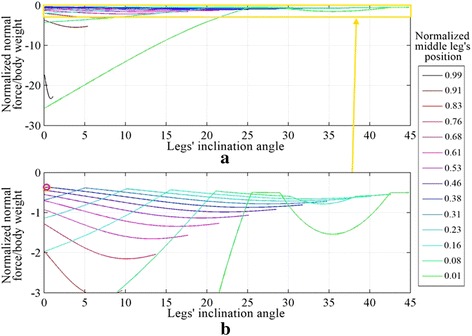
**a** The maximum adhesion required by the robot at different legs’ inclinations and different middle leg’s position. **b** zoomed in view of (**a**). The *red circle* in (**b**) shows the farthest middle leg’s position to the hind leg that improves with backward inclination of the middle leg

The maximum adhesion force required for all of the configurations is constructed by combining the three sub-figures of Fig. 7, and their plot is shown in Fig. 8. The lower plot in Fig. 8 is a zoom of the upper plot in Fig. 8.

From Fig. 8, the backward inclination of the legs improves the adhesion requirement when the middle leg’s position is below 0.38 (see red circle in Fig. 8b). The backward legs’ inclination for any other position will cause an increase in the required force. The effect of legs’ inclination for different height to length ratios has the same effect as the investigated structure with 1:2 height to length ratio; with the exception that the point that improves with the backward legs inclination is varied to be between 0.38 and 0.41 for the range of heights considered in Part 1.

An optimization using Genetic Algorithms (GA) is carried out to find the optimal configuration for the structure. It is assumed that the distance between the front and hind tips of the legs and the height are kept fixed at 200 and 100, respectively. The variables are, therefore, the position of the middle leg *d*_h_ and the inclination of all of the legs [angles of the legs are given by Eqs. (1) and (2)] and the range of *θ*_inc_ is from 0° to 45°. The investigated optimization problem is:

3 $$\mathop {\hbox{min} }\limits_{{d_{\text{m}} , \theta_{\text{inc}} }} \hbox{max} \left( {F_{\text{yh}} ,F_{\text{ym}} ,F_{\text{yf}} } \right)$$

The optimal configuration found is when the inclination of the front and the middle legs is at the maximum front, at −45°, and the hind leg is at the maximum from the perpendicular, at −135°. This result is similar to that found by Yasong et al. [2] with the assumption that the structure has infinite stiffness.

### Optimal geometry

**Fig. 9:**
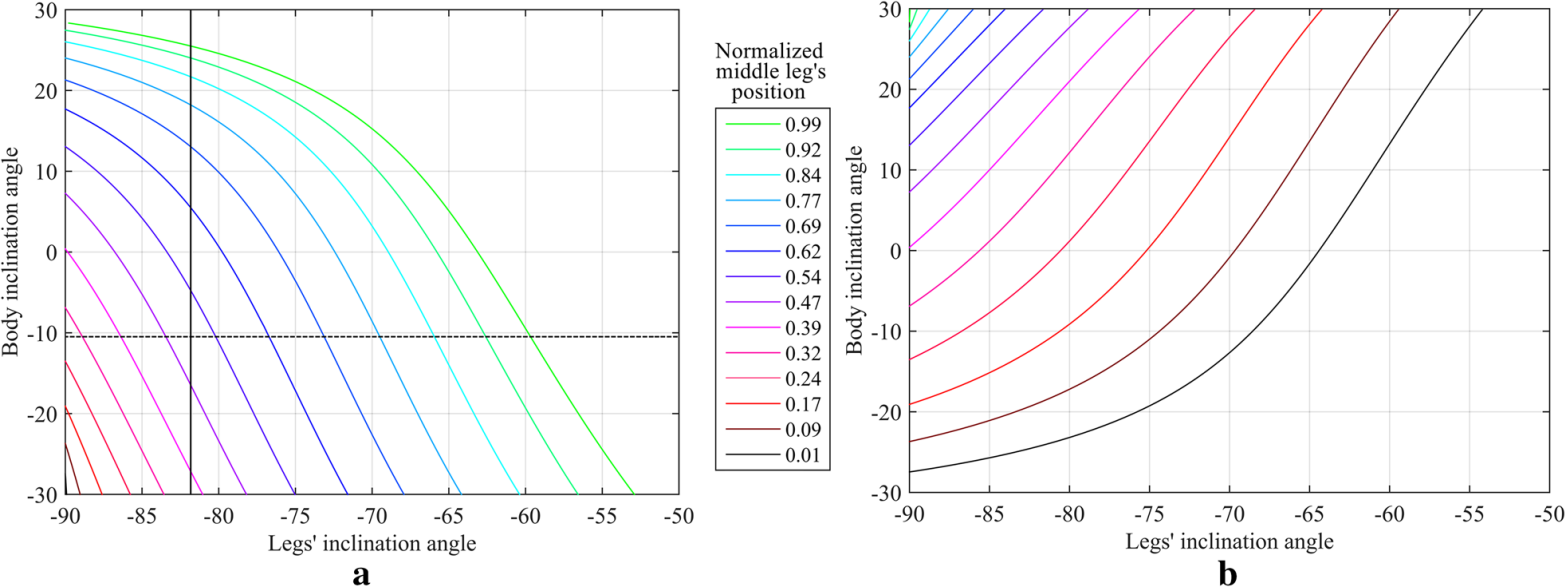
Optimal body–legs inclination curves for a number of middle legs positions when **a** the middle leg is inclined forward, **b** the middle leg is inclined backward

An infinite number of configurations of the robot can be identified when different inclinations of both the body and the legs are considered. The space of the optimal configurations is summarized in Fig. 9. For the sake of clarity, this figure shows optimal curves when all the legs have the same absolute value of inclination, and a body inclination range between −30° and 30°. In Fig. 9a, the front and the middle leg points forward whereas the hind leg points backward. In Fig. 9b, the front leg points forward whereas the middle and hind legs point backward. Points on each curve of these figures represent equally optimal configurations from the perspective of minimizing the needed maximum adhesion for the robot to stay on a vertical surface. It should be noted that the maximum required adhesion decreases as the middle leg is positioned closer to the front leg; in fact, the optimal configuration is the one that has the middle leg aligned with the front leg. In Fig. 9, points on the green curve at the normalized position 0.99, therefore, yield the smaller maximum adhesion required for the robot to adhere to vertical surface than any other point presented in this figure.

**Fig. 10:**
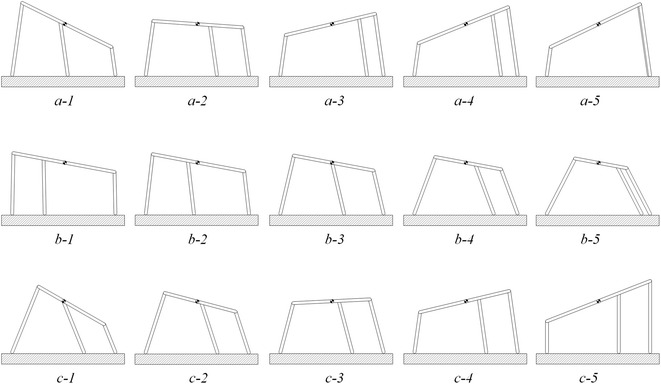
Few optimal configurations. Subplots *a-1* to *a-5* are different optimal configurations along the *vertical solid black line* of Fig. 9a when legs are inclined at −82°. Subplots *b-1* to *b-5* are different optimal configurations along the *horizontal dashed line* of Fig. 9a when the body inclination is −10.5°. Subplots *c-1* to *c-5* are different optimal configurations at middle leg’s position of 0.69 in Fig. 9a

A few optimal configurations chosen from Fig. 9, for different positions of the middle leg and different body and leg inclinations, are shown in Fig. 10. Specifically, Fig. 10a shows five different optimal configurations when the inclination of the legs was kept constant (configurations shown in Fig. 10a were obtained by intersecting the curves of Fig. 9a with the vertical black solid line shown in this latter figure). Figure 10b shows five different optimal configurations when the body inclination was kept constant (configurations shown in Fig. 10b were obtained by intersecting the curves of Fig. 9a with the horizontal dashed black line shown in this latter figure). Figure 10c shows five different optimal configurations when the distance between the tips of the legs on the vertical surface was kept constant (configurations shown in Fig. 10c were obtained by intersecting the blue curve of Fig. 9a with middle leg’s position of 0.69—see legend of Fig. 9a).

## Model verification: structural analysis for ants’ stance on vertical surfaces

**Fig. 11:**
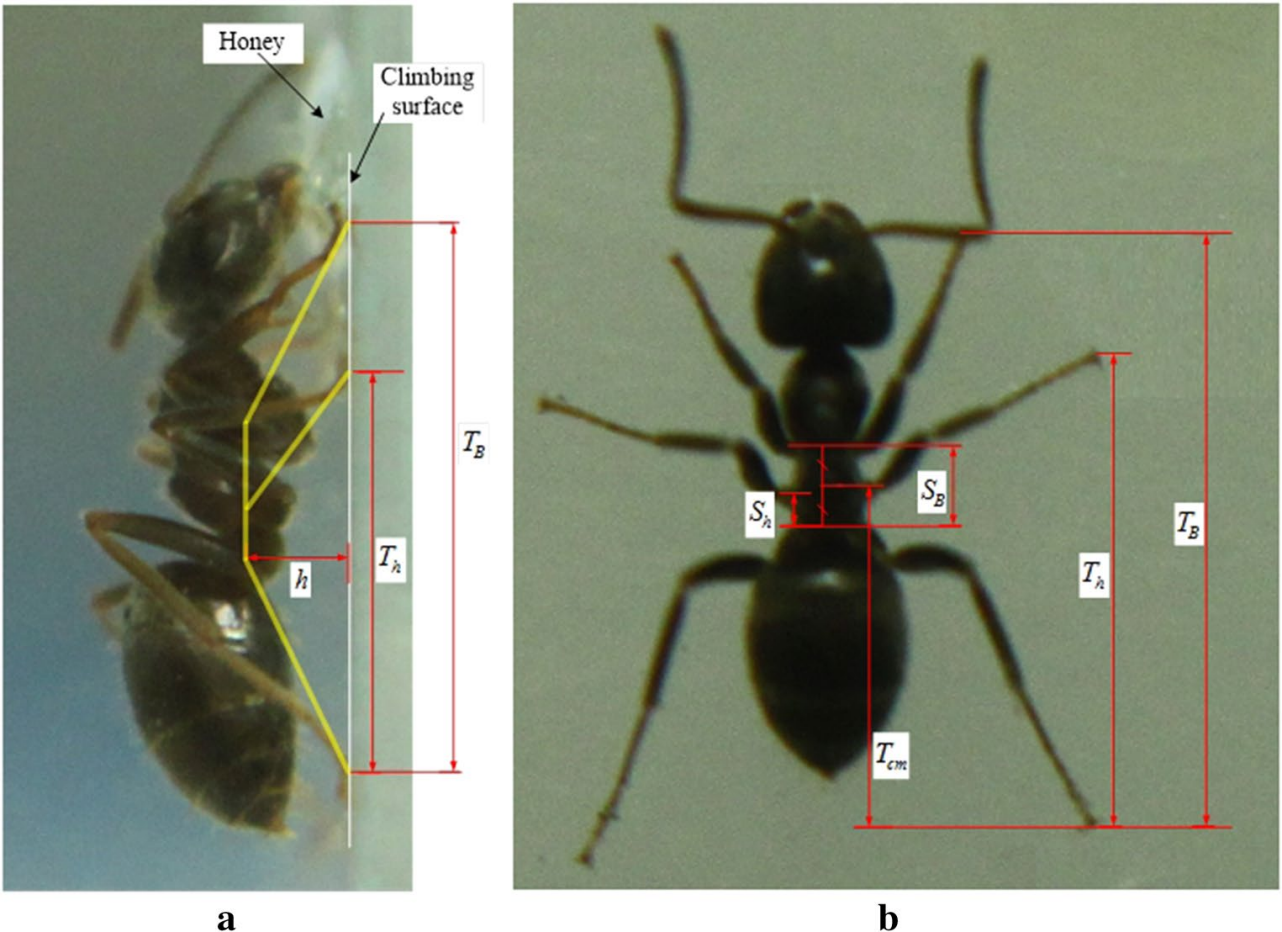
The parameters measured from the ants, **a** lateral view, **b** dorsal view

To verify our assumptions and calculations, the developed model and obtained FEM results are used to investigate the stance of the ants when loitering on vertical surfaces. Ants are chosen for this study because they are good climbers and they have a configuration similar to the studied six-legged robot. The ant along with the equivalent robot structure, highlighted in yellow, is shown in Fig. 11a. The measured parameters used to simplify the structure of the ants to match the robot’s are shown in Fig. 11a, b, where:

- *T*_B_ is the distance between the front and hind legs’ tips.
- *S*_B_ and *T*_cm_ are the body’s length and position, respectively.
- *S*_h_ is the position of the middle leg’s coxa.
- *T*_h_ is the position of the middle leg’s tip.
- *h* is the height.
- *r*, not shown in Fig. 11, is the body and legs’ thickness.

The ants are photographed when they are standing still to feed off honey drops on a surface of vertically mounted plexi-glass. In total 150 ants are used, where 91 ants are photographed from either the top or the bottom, similar to that shown in Fig. 11b, and 59 ants are photographed from the side, similar to that shown in Fig. 11a.

In both of the photographed positions, i.e., from above and the side, the following parameters are measured: *T*_B_, *S*_B_, *T*_cm_, *S*_h_, and *T*_h_ (see Fig. 11). The height is measured only using the photos captured from the side. The photos captured from above are used to make sure that the ants are standing up vertically with an angle range of ±20° from vertical, since a change in the orientation of 20° would decrease the force pulling an ant to the back, due to gravity, by only ±6 %.

**Fig. 12:**
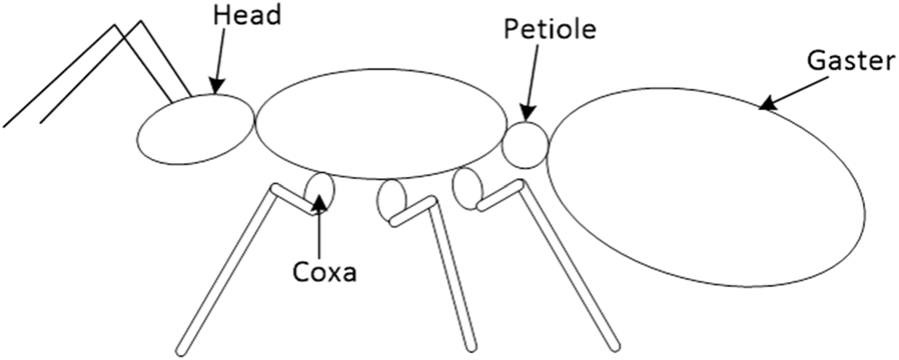
The different parts of ant

The length of the body *S*_B_ is measured as the distance between the front and the hind legs’ first segments, called coxas, see Fig. 12. The position of the body *T*_cm_ is measured as the distance from the center of the body’s length to the hind leg’s tip. The measurement of the middle leg’s coxa position *S*_h_ is represented as the distance between the coxa of the hind leg and the coxa of the middle leg. When the ratio equals 0, then the middle leg’s coxa is at the same position as the hind leg’s coxa. The position of the coxa approaches the front leg’s coxa as the value of *S*_h_ approaches 1.

The position of the middle leg’s tip *T*_h_ is the distance between the front and the hind legs’ tips. The height of the robot *h* is presented as the distance from the petiole to the climbing surface, which is considered the point of force application. The petiole is chosen because it is the point by which the gaster (which has a large weight ratio) is attached to the body, see Fig. 12. The average thickness of the legs *r* is measured graphically. Specifically, the thickness of each segment of the leg is averaged over the length of the leg. The thickness is calculated using the following equation:

4 $${\text{Thickness}} = \frac{{\mathop \sum \nolimits_{i = 1}^{n} \left( {{\text{segment}}\,\,{\text{thickness}} \cdot {\text{length}}\,\,{\text{of}}\,\,{\text{that}}\,\,{\text{segment}}} \right)}}{{{\text{length}}\,\,{\text{of}}\,\,{\text{the}}\,\,{\text{leg}}}}$$

where $n$ is the number of segments in the leg.

All of the measurement data are presented as ratios to overcome the variation in ants’ sizes. *T*_B_ is considered as a measuring unit for all of the measurements, i.e., *T*_B_ = 1, except for *S*_h_ which is measured with *S*_B_ as the measuring unit.

Assuming a robot with *T*_B_ = 200, which has the same *T*_B_ as the robots investigated earlier, the parameters of the robot equivalent to the ants’ geometry are calculated and shown in Table 1. The weight of the ant is considered to be a unit to facilitate the representation of the force to be a fraction of the overall weight.

**Table 1:** Parameters measured experimentally and the equivalent values used in calculations

Parameter	Experimentally	Value
*T*_B_	1	200
*T*_h_	0.62	124
*h*	0.1439	28.78
*S*_B_	0.169	33.8
*S*_h_	0.358	12.1
*T*_cm_	0.616	123.25
*r*	0.022	4.4

The developed mathematical model presented earlier is used to compute the maximum required adhesion force for *S*_h_ and *T*_h_ while keeping all the other parameters fixed. The value of *S*_h_ is varied within the full range of 0–1, with the middle leg’s coxa coinciding with the hind leg’s coxa when *S*_h_ = 0, and coinciding with the front leg’s coxa when *S*_h_ = 1. Similarly, the value of the middle leg’s tip position *T*_h_ is considered to be 0 when the tip is aligned with the hind leg’s tip, and 1 when the middle leg’s tip is aligned with the front leg’s tip, i.e., the distance to the hind leg’s tip is 200.

**Fig. 13:**
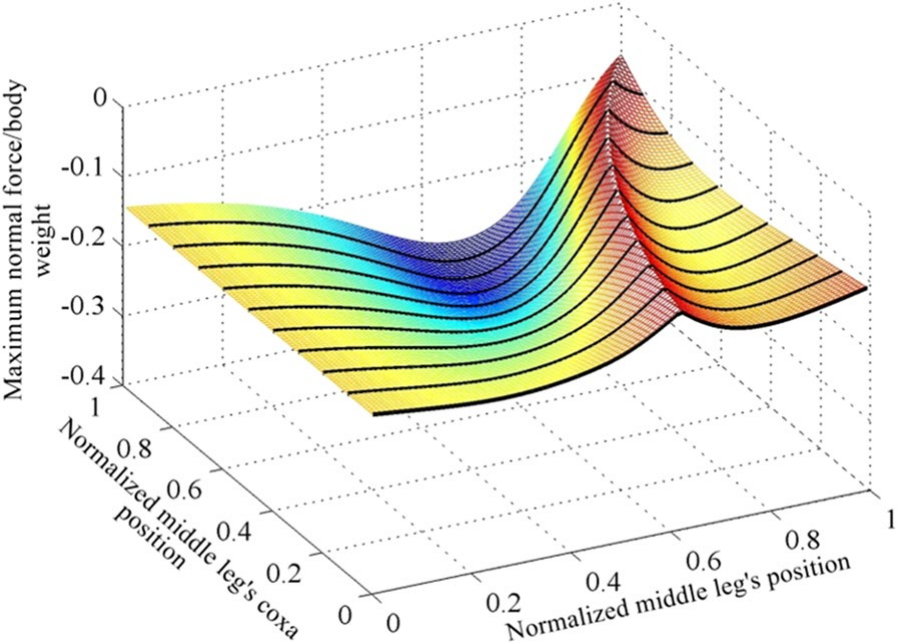
The adhesion requirement for different middle leg’s coxa and tips positions

The maximum required adhesion at different values of *T*_h_ and *S*_h_ for a unit force representing the weight at the center of mass is shown in Fig. 13. It is noted that the smaller the distance between the middle and hind legs’ coxas, the less is the maximum required adhesion force. From the photos of the ants, the middle and the hind legs’ coxas are as close as possible to each other; in fact the mid and the hind coxas are in contact with each other. The ratio of the distance between the center of the middle leg’s coxa and the center of the hind leg’s coxa to the distance between the centers of the hind and the front coxas is 0.358.

**Fig. 14:**
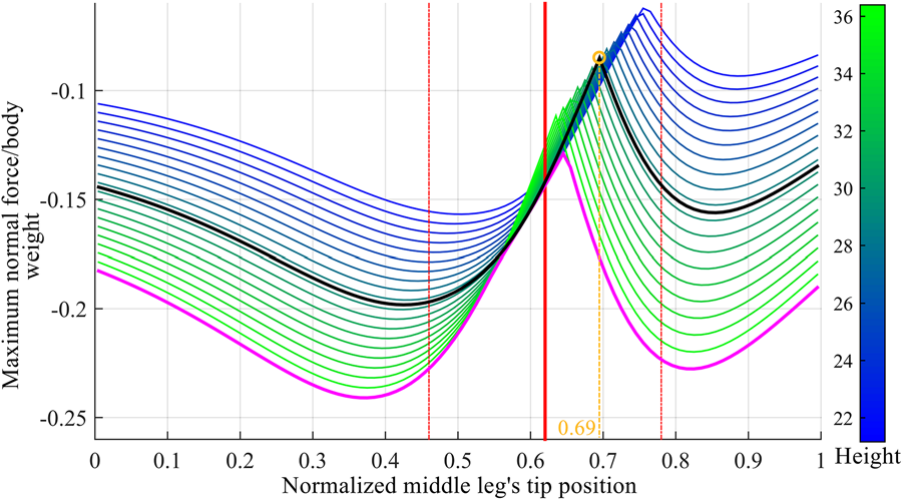
The normal force requirement at different heights, in colors varies from *green* to *blue*. The *black line* is the normal force requirement at the median height, the middle leg’s tip position obtained experimentally is shown in *solid red line* and its standard deviation in *dashed red lines*. The maximum adhesion required at the median height is marked in *yellow color*

Figure 14 shows the maximum normalized adhesion force when a ratio between *S*_B_ and *S*_h_ of 0.36 is considered. The different lines in this figure represent forces for the different heights the ants had in the recorded images. The black line in Fig. 14 shows the force for the median height of the ants. The position of the middle leg’s tip used by the ants is plotted as a solid red line, with its standard deviation plotted as a red dashed line. The optimal position for the tip is at 0.69, which corresponds to the highest point on the black curve in Fig. 14, is not far from that found experimentally with the ants. In fact, the ants’ average position of the middle leg’s tip is different by only 8 % away from the calculated optimal tip position.

Curves of maximum normal force for a range of heights for the robot are shown in Fig. 14 and are colored with shades varying from blue to green. The different heights are added to this figure to analyze the effect of changing the height; the authors in fact noticed that ants change their body height while loitering. The magenta line in Fig. 14 is obtained by intersecting all the curves in this figure and considering their lower values for each middle leg’s tip position. It, therefore, represents the maximum normal adhesion requirement for each position of the middle leg’s tip. It should be noted that the maximum value of this line is close to the averaged middle leg position of the ants (vertical red line in Fig. 14 at 0.62 normalized middle leg’s position). The maximum of that curve represents the position of the middle leg’s tip that experiences the minimum adhesion force requirement for the different heights. That point is different by only 2.6 % from the middle leg position used by the ants. It is interesting to note that the position of the middle leg used by ants to minimize the maximum adhesion required to adhere to vertical surfaces is 0.62, which is very close to the golden ratio often found in nature [27–29].

**Fig. 15:**
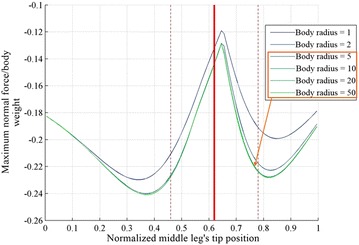
The maximum normal force requirement for different body thicknesses at different middle leg’s position. The considered body thicknesses are 1, 2, 5, 10, 20 and 50 times the thickness of the legs, they are highlighted in gradient colors from *blue* to *green*. The body thicknesses of 5 and higher overlap. The *solid red line* is the middle leg’s tip position obtained experimentally and the *dashed red lines* are the standard deviation

The thickness of the body used in the calculations is approximated to be 5 times the thickness of the legs. The effect of body thickness on the curve of the maximum adhesion and the position of the minimum adhesion point is shown in Fig. 15. Although the curves are not identical between the considered thicknesses, the point that requires the minimum adhesion is still the same for the different radius values.

## Discussion

The presented results yield the following guidelines to design an efficient robot loitering on a vertical surface:

- For a fixed body length, all the legs of the robot should be inclined outwards, that is they should be extended as far as possible to increase the distance between the front and the hind tips of the legs.
- The optimal position for the middle leg is to be as close as possible to the front leg and the inclination should be forward for a middle leg’s position between approximately 0.4 to 1, and backward otherwise.
- The optimal result is found when the hip joints for the legs coincide and the middle leg’s tip position is at the same position as the front leg’s; this finding is confirmed though an optimization performed using GA [see Eq. (3)].
- Depending on the height to length ratio, tilting the body mostly improves the adhesion requirement for the robot.
- The optimal body and legs inclination for any middle leg’s position could be chosen from Fig. 9.

The steps used to investigate the effect of the different parameters on the structure of the robot are used to analyze the stance the ants use on vertical surfaces. Analytically, it is found that the closer the middle leg’s coxa is to the hind leg’s coxa the less the adhesion force is over the entire range of the middle leg’s tip position. Interestingly, the coxas of the middle and the hind legs are touching each other in ants, that is they are as close as their size allows. The distance from the center of the coxa of the middle leg to the center of the coxa of the hind leg is 33 % of the body length on average on the collected ants. The optimal middle leg’s tip position is at approximately 61 % of the distance between the tips of the hind and front legs pointing forward.

## Conclusion

In this work, an investigation of the effect of the inclination of the body and legs on the minimal adhesion requirement for a climbing robot to adhere to a vertical surface is investigated. It is found that the optimal configuration to minimize the force required to adhere to a vertical surface is when the front and the middle legs are inclined forward and their tips overlap. Also, tilting the front of the body reduces the required adhesion force. The structural model used to investigate the effect of the different parameters on the adhesion requirements is used also to explain the positioning of the tip of the middle leg the ants use to stand on vertical surfaces; the stance that ants use minimizes the maximum adhesion force over the full range of the middle leg’s tip position. The model developed in this work is applicable to six-legged robots. Similar to the analysis performed to investigate the ants’ stance, different robotic structures can be investigated following the same procedure used in this article.
